# Value of cardiovascular magnetic resonance in patients with suspected inflammatory pericarditis

**DOI:** 10.1186/1532-429X-17-S1-P349

**Published:** 2015-02-03

**Authors:** Inês Cruz, Giuseppe Muscogiuri, Peter Sinnaeve, Steven Dymarkowski, Massimo Imazio, Jan Bogaert

**Affiliations:** 1 Imaging and Pathology, KU Leuven - University of Leuven, Leuven, Belgium; 2 Cardiovascular Diseases, KU Leuven - University of Leuven, Leuven, Belgium; 3 Cardiology, University of Torino, Torino, Italy

## Background

Although the diagnosis of inflammatory pericarditis is usually presumed on a combination of clinical signs and noninvasive testing, especially echocardiography, the presence and extent of pericardial inflammation as such cannot be visualized. Alternatively cardiovascular magnetic resonance imaging (CMR) has shown appealing to study the pathologic pericardium. In this study we sought to explore the findings and the diagnostic value of cardiovascular magnetic resonance imaging (CMR) in patients with clinically suspected inflammatory pericarditis.

## Methods

Review of CMR data between January 2010 and December 2013 yielded 42 patients (51±16y, 31 males) with clinical suspicion of inflammatory pericarditis. In 21 patients (50%), it was their first presentation, the others presented a recurrence.

## Results

Transthoracic echocardiography (TTE), performed at 3.6±5.6 days after symptom onset, showed a pericardial effusion in 14/42 patients (33.3%). CMR, performed 7.1±5 days after symptom onset, showed a pericardial thickness of 2.3±1.4mm (max 6.4mm). In 11 patients (26%) a pericardial effusion was present. On T2-weighted imaging pericardial edema was present in 23/40 patients (58%) while pericardial enhancement was found on late gadolinium-enhanced (LGE) images in 39/42 patients (93%). Six patients showed concomitant subepicardial myocardial enhancement. Real-time cine CMR showed inspiratory septal flattening/inversion in 11 patients. Patients with pericardial thickness ≥4mm (n=8), more frequently presented with fever (p=0.026), had increased C-reactive protein values (p<0.0005), increased signal intensity of pericardial enhancement (p=0.016), and increased septal shift (p=0.001). No significant differences with respect to clinical presentation and CMR findings were found between patients with first and recurrent pericarditis. Seven patients presented recurrence during follow-up (mean 414 days). CMR findings yielded no significant prognostic value on events occurrence.

## Conclusions

In patients with clinical suspicion of inflammatory pericarditis, CMR is a valuable tool to appreciate the presence and severity of pericardial inflammation. As such, this technique may be helpful in doubtful cases and to rule out concomitant myocardial involvement.

## Funding

No funding.

**Figure 1:**
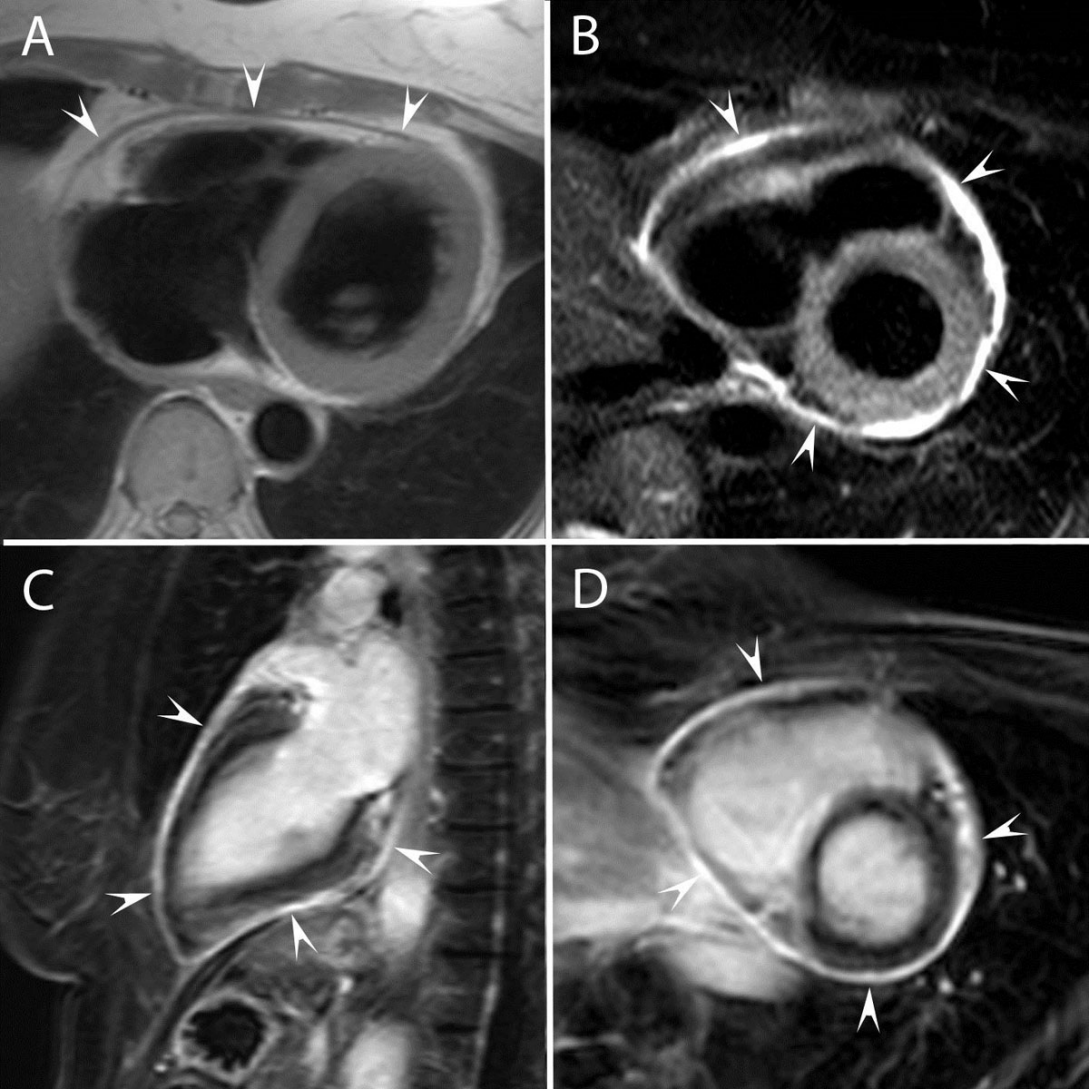
**Recent history of acute idiopathic pericarditis, presenting recurrent complaints of chest pain and elevated CRP values. Initial TTE showed minimal pericardial effusion.** Axial T1-weighted image (A), short-axis T2-weighted image (B), LGE-image in vertical long-axis (C) and short-axis (D). Minimally thickened pericardial layers (2.5 mm thickness) with SI similar to that of myocardial tissue on T1-weighted imaging (arrowheads, A). High SI on T2-weighted imaging (arrowheads, B), and strong pericardial on LGE-imaging (arrowheads, C,D). No evidence of residual pericardial fluid.

